# Acute pulmonary alveolar proteinosis due to exposure to cotton dust

**DOI:** 10.4103/0970-2113.56355

**Published:** 2009

**Authors:** Gurcharan Singh Thind

**Affiliations:** *Department of TB and Chest Diseases, Medical College Hospital, Amritsar, India*

**Keywords:** Alveolar macrophage, bronchoalveolar lavage, cotton dust, granulocyte macrophage colony-stimulating factor, pulmonary alveolar proteinosis

## Abstract

Secondary pulmonary alveolar proteinosis (PAP) is rare but may occur in association with malignancy, certain infections, and exposure to inorganic or organic dust and some toxic fumes. This case report describes the second recorded case of PAP due to exposure to cotton dust. A 24-year-old man developed PAP after working as a spinner for eight years without respiratory protection. He was admitted as an emergency patient with very severe dyspnea for four months and cough for several years. Chest X-ray showed bilateral diffuse alveolar consolidation. He died 16 days later, and a diagnosis of acute pulmonary alveolar proteinosis was made at autopsy. The histopathology demonstrated alveoli and respiratory bronchioles filled with characteristic periodic acid Schiff-positive material, which also revealed birefringent bodies of cotton dust under polarized light. Secondary PAP can be fatal and present with acute respiratory failure. The occupational history and characteristic pathology can alert clinicians to the diagnosis.

## INTRODUCTION

Pulmonary alveolar proteinosis (PAP) is a rare disease, which is characterized by accumulation of lipoproteinaceous material [periodic acid Schiff (PAS)–positive material] in the alveoli.[1–4] PAP is now recognized to occur in three distinct forms: Congenital, acquired idiopathic, and secondary. Secondary PAP develops in conditions where there is impaired function or reduced number of alveolar macrophages (AMs). It is associated with some hematological cancers, immunodeficiency disorders, inhalation of inorganic dusts and toxic fumes, and certain infections.[2–4] Kosacka *et al.* recently reported one case of secondary PAP after exposure to cotton and linen dust.[5] This case report represents the second recorded case of PAP due to heavy exposure to cotton dust. The illness was fatal, and interestingly the histopathology of acute pulmonary alveolar proteinosis resembled acute silico-proteinosis.

## CASE REPORT

A 24-year-old man was referred to the emergency department with a complaint of dyspnea of four months' duration, which was worse for the last one week. This was associated with productive cough for the same duration. There was no history of fever, chest pain, wheeze, anorexia, and weight loss. There was history of cigarette smoking of one pack per day for the last 10 years. He was a spinner in a cotton spinning and weaving mill for eight years and was exposed to heavy cotton dust without using respiratory protection. There was no history of exposure to any other dust.

**Figure 1:**
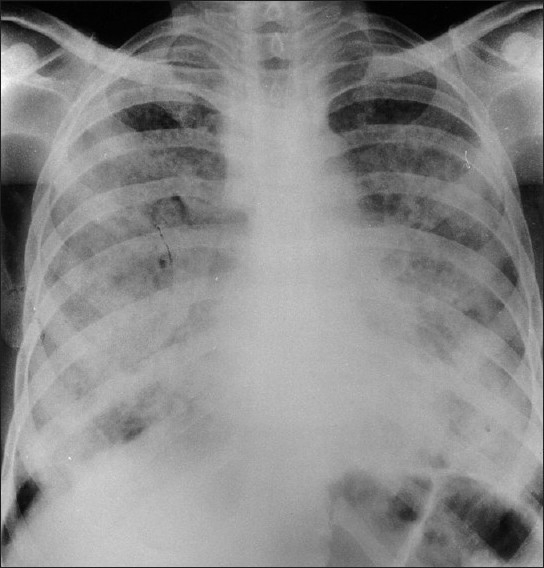
Postero-anterior view of chest radiograph showing nonspecific extensive bilateral patches of lung opacities, more marked in the lower zones

On examination, his pulse rate was 98 beats per minute, respiratory rate was 32 breaths per minute, blood pressure was 125/75 mmHg, and temperature was 39.5°C. The patient was also noted to be cyanosed with gross clubbing. Respiratory examination revealed bilateral diffuse crepitations and rhonchi. Chest radiograph showed diffuse bilateral alveolar opacification, more marked in the middle and lower zones [Figure 1]. The possibilities considered were COPD with super-added infections, pulmonary tuberculosis, alveolar cell carcinoma, diffuse fibrosing alveolitis, and PAP with secondary infections due to bacteria or fungi. The results of liver and renal function tests, complete blood count, and urine analysis were normal. Sputum tested negative for acid-fast bacilli (AFB) by Ziehl Neelson stain (done daily) and for malignant cells and fungi, and grew no organism on culture. Spirometry could not be done. The patient was managed with antibiotics, bronchodilators, and oxygen therapy, following which he became afebrile but his general condition and dyspnea got worse; and he died 16 days after admission. Parts of lung were procured from postmortem for histopathological examination.

## HISTOPATHOLOGY

**Figure 2:**
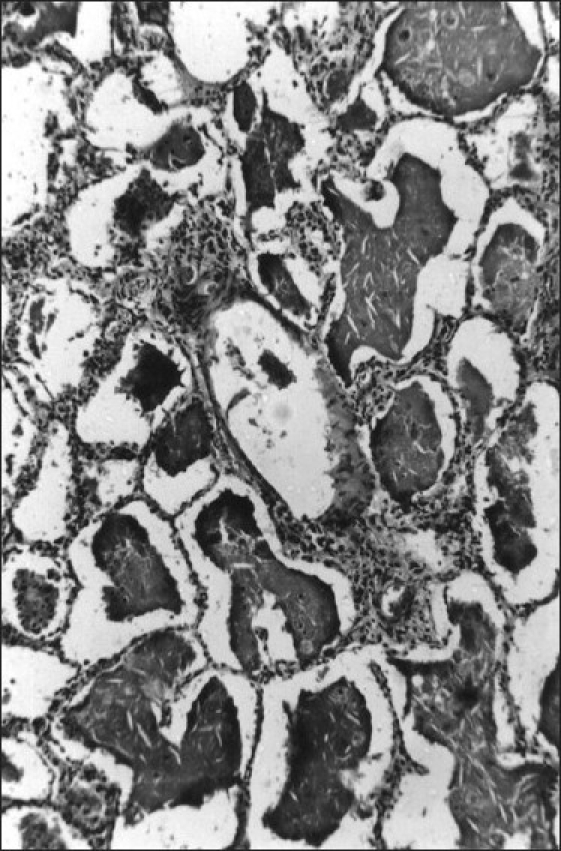
Histopathological picture of lung section showing alveoli filled with eosinophilic amorphous material; the intra-alveolar material shows cleft-like spaces and foamy macrophages. The lining alveolar cells were swollen. Parenchymal architecture remained intact (H and E, ×50)

**Figure 3:**
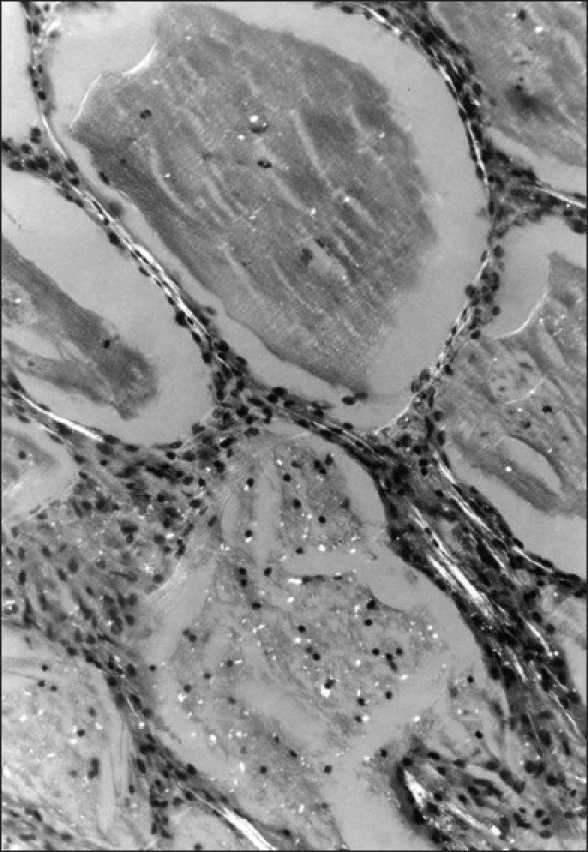
Lung section photograph under polarized light showing birifringent bodies of cotton dust in the alveoli (H and E, ×250)

Histopathology showed alveolar spaces and respiratory bronchioles filled with eosinophilic PAS-positive material typical of PAP, with cleft-like spaces scattered throughout. The lining alveolar epithelial cells were swollen, and some desquamated cells were included in the material filling the alveolar spaces. The alveolar septa and the walls of the respiratory bronchioles were slightly thickened due to infiltration by lymphocytes and histiocytes [Figure 2]. Under polarized light, a number of birefringent bodies were seen due to the presence of cotton particles [Figure 3].

## DISCUSSION

This case represents the second recorded case of PAP due to cotton dust exposure. While most cases of PAP are considered idiopathic, this case emphasizes the importance of an exposure history in assessing patients with diffuse respiratory disease. It also demonstrates the value of histopathology in confirming the diagnosis, as well as the fact that PAP can cause fatal respiratory failure.

In recent years, great progress has been made in understanding the pathogenesis of this rare and puzzling disease, and toward the development of its treatment. The role of granulocyte macrophage colony-stimulating factor (GM-CSF) dysfunction in congenital and acquired idiopathic PAP is now recognized. Secondary PAP develops in association with many conditions, causing functional impairment or reduced number of alveolar macrophages. The cotton dust exposure may either impair alveolar macrophage function or stimulate surfactant production to such an extent that it overwhelms clearance mechanisms, and PAP develops.

About 70% of patients of PAP, including the patient in the present case, were smokers. Smoking may act as a predisposing factor since it reduces the ciliary activity and hence clearance of the particulate matter from the lung.

Some cases of PAP may have low-grade fever, but high degree of fever usually indicates the presence of a complicating infection.[2,3] The patient in the present case had high fever, 39.5°C. He became afebrile with antibiotic treatment, indicating the presence of secondary infection, even though we could not grow any organism on culture of sputum.

Although definite diagnosis of PAP has usually been made at autopsy or open lung biopsy, recent observations show that examining the cytology of bronchoalveolar lavage (BAL) may play a very important role in the diagnosis of PAP.[4]

Treatment of secondary PAP involves the treatment of the underlying condition; for example, when it is associated with hematologic cancer, successful chemotherapy or bone marrow transplantation corrects the associated PAP.[3] In many cases of secondary PAP, BAL can be used with good therapeutic effect. Acquired or idiopathic PAP has been successfully treated since 1960 by using BAL, and this procedure is still the standard treatment.[2,3,6] Recently GM-CSF as injection or aerosol therapy has shown promising results.[2,3] The patient in the present case was not treated for PAP as the diagnosis was made at autopsy.
